# Type 2 diabetes-associated single nucleotide polymorphism in *Sorcs1* gene results in alternative processing of the Sorcs1 protein in INS1 β-cells

**DOI:** 10.1038/s41598-019-55873-6

**Published:** 2019-12-19

**Authors:** Belinda Yau, Zachary Blood, Yousun An, Zhiduan Su, Melkam A. Kebede

**Affiliations:** Charles Perkins Centre, School of Life and Environmental Sciences, Faculty of Science, The University of Sydney, Sydney, New South Wales Australia

**Keywords:** Endocrinology, Protein transport

## Abstract

A threonine-to-Isoleucine (Thr_52_Ile) mutation within the pro-domain of the Sorcs1 gene was positionally cloned as the gene underlying a quantitative trait locus that affects fasting insulin levels in mice. In humans, genome-wide association studies and linkage studies have shown that SORCS1 is associated with diabetes and all of diabetes complications. We have recently shown that deletion of *Sorcs1* in mice made obese with the *leptin*^*ob*^ mutation results in diabetes and an insulin granule stability defect. This present study investigates the functional consequence of the *Sorcs1* Thr_52_Ile mutation in the rat INS1 β-cell line expressing either the wildtype or mutant Sorcs1 allele. We find that *Sorcs1* Thr_52_Ile mutation is associated with increased basal insulin secretion, reduced glucose-stimulated insulin secretion and decreased insulin content in INS1 cells. Moreover, expression of Thr_52_Ile causes differential processing of the Sorcs1 protein resulting in the formation of an additional 90 kDa mutant form of the protein. The mutant form of the protein is localised to the ER, retains its pro-domain, and concurrently reduces expression of the functional mature 130 kDa Sorcs1 protein. These findings provide a mechanistic clue to why this specific allelic variation in *Sorcs1* was associated with reduced insulin levels and type 2 diabetes.

## Introduction

Sorcs1 is a member of the vacuolar protein sorting -10 (VPS10) family of proteins. VPS10, when identified in yeast, was described as a receptor expressed in the trans-Golgi-network (TGN) that trafficks its cargo (the vacuolar hydrolase pro-carboxypeptidase-Y) from the TGN to the endosome^1^. In mammals, there are five members of the VPS10 protein family; Sortilin, SorLA, and Sorcs1-3. Sortilin and SorLA are well-studied and have been shown to play a role in intracellular protein trafficking and sorting, suggesting that all VPS10 proteins may play a similar role.

Sorcs1 is a type I transmembrane protein that comprises an N-terminal signal peptide, a pro-domain, a Vps10 domain (Vps10-D), a leucine-rich polycystic kidney disease domain, a transmembrane domain, and a short C-terminal cytoplasmic tail^2^, with alternatively spliced isoforms of the protein exhibiting differential subcellular localisation^3,4^. The conserved and characteristic Vps10-D of Sorcs1 and its family members contains a β-propeller forming a tunnel via which soluble ligands can interact^5^ and be transported between subcellular compartments^6^.

The link between *Sorcs1* mutation and pathophysiology was first implicated in late-onset Alzheimer’s disease^7^, in which amyloid precursor protein transport and subsequent amyloid-β secretion are affected. *Sorcs1* overexpression correlated with increased amyloid-β secretion, while a mutation in *Sorcs1* at the cytoplasmic tail domain increased amyloid-β generation in human embryonic kidney cells, demonstrating a specific role for *Sorcs1* in secretory protein retention and anterograde trafficking between the Golgi and the plasma membrane^8,9^.

Using a forward genetics approach employing the diabetes-resistant C57BL/6J (B6) and the diabetes-susceptible Black and Tan Brachyury (BTBR) mouse strains, introgression of the *ob/ob* allele identified quantitative trait locus for type 2 diabetes on chromosome 19 in the F2 generation^10^. Localisation of this locus in subcongenic BTBR mice expressing both the *ob/ob* allele and segments of B6 chromosome 19 revealed a single nucleotide polymorphism in the *Sorcs1* gene (threonine-to-isoleucine substitution at amino acid 52 (Thr_52_Ile)), associated with the diabetic phenotype^10^. Mice homozygous for the B6 *Sorcs1* allele exhibited fasting hyperglycaemia, reduced fasting plasma insulin levels, and irregular pancreatic islet morphology^10^. Subsequently, SORCS1 was identified in several human genome-wide association studies to be associated with diabetes^11,12^ and all of diabetes complications; neuropathy, hypoglycemia unawareness, retinopathy and nephropathy^13^.

In a follow up study, we generated mice with a global deletion of the *Sorcs1* gene. When made obese with the *leptin*^*ob*^ mutation, the mice were severely diabetic and the pancreatic β-cells had a severe deficiency in insulin secretory granules. We concluded that Sorcs1 is involved in the trafficking and biogenesis of the insulin secretory granules^14^.

While the phenotype of Sorcs1 knock out mice provided an opportunity to study the consequence of a complete *loss-of-Sorcs1*, it does not necessarily provide true insight into the functional consequences of the non-synonymous Thr_52_Ile *Sorcs1* variants associated with type 2 diabetes. The objective of this study was to investigate the consequences of *Sorcs1* Thr_52_Ile in an *in vitro* model employing the well-established INS1 β-cell line. Under stable doxycycline inducible expression of either the wildtype BTBR *Sorcs1* allele (*wtSorcs1)*, or the mutant B6 *Sorcs1* allele (*mutSorcs1)*, differential processing of the Sorcs1 protein was observed. *MutSorcs1-*expressing cells demonstrated the formation and endoplasmic reticulum (ER) retention of a 90 kDa mutant pro-protein, which exhibited an extended half life and resulted in reduced total expression of the mature Sorcs1 protein. Functionally, this reduction was associated with increased basal insulin secretion, blunted glucose-stimulated insulin secretion and reduced total insulin content. As *Sorcs1* mutation drives an obesity-regulated diabetic phenotype in animals, these findings have significant implications for the human-associated gene variants and substantiates its role as a genetic contributor to type 2 diabetes.

## Materials and Methods

### Cell culture

Glucose-responsive INS1 832/13 (INS1) rat β-cell line were stably transfected with wild type or mutant *Sorcs1* cDNA, with a myc tag at the C-terminal, using the Tet-On system to generate doxycycline-inducible *Sorcs1-*expressing cells. Cells were cultured in RPMI 1640 medium supplemented with 10% FBS, 2 mM L-glutamine, 1 mM sodium pyruvate, 50 mM beta-mercaptoethanol, 10 mM HEPES, 1% penicillin-streptomycin, at 37 °C in 5% CO_2_. *Sorcs1* expression was induced by addition of 2.25 µM doxycycline hyclate (Sigma, D9891) into culture media for 18 h at 37 °C (or 15 °C and 20 °C for temperature trapping experiments).

### Generation of doxycycline inducible stable cell lines

The T-REx^Tm^ system from Invitrogen was used to generate the doxycycline-inducible Sorcs1 wildtype and mutant stable cell lines according to the manufacturer’s instructions. Briefly, INS1 832/13 cells, which expresses very low levels of endogenous Sorcs1, were first transfected with pcDNA™6/TR plasmid, which encodes the Tet repressor (TetR) under the control of the human CMV promoter, with Lipofectamine 2000 and selected with blasticidin to generate the TetR stable cell lines. A clonal stable cell line that expresses the highest levels of TetR was then used to transfect linearized pCDNA4TOMycHisA-myc-*Sorcs1*-WT or pCDNA4TOMycHisA-myc-*Sorcs1*-WT expression vectors and selected with zeocin.

### Cell protein extraction and processing

Whole cell lysates (WCL) were obtained from cells using RIPA buffer (Merck, 20-188) containing cOmplete protease inhibitor cocktail (Roche, 4693159001), sonication using a tip probe for 20 s, and centrifugation at 14 k xg for 10 min. Protein quantification was performed using a BCA protein assay (Pierce, 23225). For disulphide bond reduction, WCL were treated with 10 mM DTT at 56 °C for 30 min, then 50 mM N-Ethylmaleimide (NEM) or iodoacetamide (IAA) was added to lysates for 40 min at RT in the dark prior to SDS-PAGE. For glycan cleavage analysis by western blot, WCL were treated with either 1250 U or 2500 U Endo H (NEB, P0702S) or 500 U PNGase F (NEB, P0709S) under denaturing conditions as per manufacturer’s protocols, after overnight induction of *Sorcs1* expression prior to analysis by SDS-PAGE. For proprotein convertase inhibition, cells were incubated in culture media containing 10 µM proprotein convertase inhibitor (Calbiochem, 537076) or DMSO control for 30 min prior to 18 h induction of *Sorcs1* expression. For protein synthesis inhibition, cells were incubated with either DMSO or 5 µM cyclohexamide for up to 2 hours prior to harvest in RIPA buffer.

### Western blotting

Cell lysates were analysed on 7.5% Tris-glycine-SDS-PAGE under reducing conditions of either 0.1 M DTT or 1% (v/v) BME, and probed with mouse anti-myc (Merck, 900000032846), mouse anti-beta-actin (Sigma, A5441) and rabbit anti-GAPDH (Santa Cruz, sc-25778) antibodies respectively.

### Immunofluorescent staining

Cells were plated onto glass coverslips, fixed with 4% PFA for 20 min at RT, washed with 0.1% BSA in PBS with 0.01% Sodium Azide. Following permeabilisation with 0.1% SDS for 5 min, cells were blocked with Serum-Free Protein Block (DAKO, X0909) for 1 h prior to overnight staining at 4 °C with mouse anti-myc (Merck, 900000032846), guinea pig anti-insulin (DAKO, A0564), rabbit anti-TGN38 (Sigma, T9826), rabbit anti-ERp72 (CST, D70D12) or rabbit anti-Calnexin (Abcam, ab22595) antibodies. Then cells were washed, incubated with fluorescent conjugated secondary antibodies (Invitrogen) for 1 h at RT in the dark, washed, then mounted onto microscope slides with Prolong Diamond Antifade Mountant containing DAPI (Thermo Fisher, P36965). Slides were imaged using a Leica TCS SP8 confocal microscope using a 100 X oil objective.

### Glucose stimulated insulin secretion assay

Cells are plated overnight in culture media, before media is replaced with HEPES Krebs Ringer buffer (KRBH) (20 mM HEPES, pH 7.4; 119 mM NaCl; 4.75 mM KCl; 2.54 mM CaCl_2_; 1.2 mM MgSO_4_; 1.18 mM KH_2_PO_4_; 5 mM NaHCO_3_), containing 2.8 mM glucose for 1 h at 37 °C for basal incubation. Cells are then stimulated with KRBH containing either 2.8 or 16.7 mM glucose for 1 h at 37 °C, and media was collected for insulin secretion determination by Insulin ELISA (Crystal Chem) or Insulin HTRF (Cisbio). Cells were washed then scraped into PBS, centrifuged at 300 xg for 5 min to pellet, then sonicated for 10 s in 50 uL of Lysis Buffer (100 mM Tris, 300 mM NaCl, 10 mM NaF, 2 mM Na) for analysis of total cell DNA concentration (Quant-iT Picogreen DNA kit, Thermo Fisher) and total cell insulin content (HTRF, Cisbio). Insulin secretion and insulin content data are both expressed relative to total cell DNA content.

### Subcellular fractionation

Whole cell protein extracts were collected by scraping cells into HEPES/EGTA buffer (5 mM HEPES, 1 mM EGTA), lysed by needle pulse by passing 15 times through a 21-gauge needle, then centrifuged at 1000 xg, 5 min and 4 °C. Supernate was layered on the top of a continuous sucrose gradient of 2 M to 0.45 M sucrose in HEPES/EGTA buffer and centrifuged in a Thermo Fisher superspeed centrifuge (A22-24 × 16 rotor) for 18 h. Sucrose fractions were collected from the top of the gradient and diluted 2:1 with reducing Laemmli sample buffer (4% SDS, 20% glycerol, 10% beta-mercaptoethanol, 0.004% bromphenol blue, 0.125 M Tris HCl) for analysis by SDS-PAGE.

### Sorcs1 immunoprecipitation

WCL was pre-cleared using 50% Protein G Sepharose beads (GE Life Sciences, 17061801) prior to immunoprecipitation. Precleared WCL was incubated with anti-myc tag antibody-conjugated agarose beads (Merck, 16-219) overnight at 4 °C, centrifuged at 1000 xg for 30 s, washed at least three times with NP-40 buffer, then twice with PBS. Beads were resuspended into 4X Laemmli sample buffer and protein was eluted with incubation at 95 °C for 10 min and analysed by SDS-PAGE.

### In-gel digestion

Protein eluate was separated in a 7.5% Tris-glycine SDS-PAGE and incubated with Coomassie brilliant blue G-250 (Sigma, B0770) for 20 min, rinsed in distilled water and destained in 10% methanol and 7% acetic acid to remove background staining. Protein bands were excised from the gel, diced into 1 × 1 mm pieces and immersed in 30% acetonitrile and 7% triethylammonium bicarbonate and incubated in a Thermomixer C (Eppendorf) for 15 min at 1000 rpm at 22 °C, until gel pieces appeared colourless. The gel was then dried for 5 min in a GeneVac EZ-2 (SP Scientific), incubated in 100 mM DTT and 100 mM NH_4_HCO_3_ at 56 °C for 30 min. Supernate was discarded, gel pieces were dehydrated in acetonitrile for 5 min, supernate removed again and gel pieces dried by GeneVac EZ-2 for 5 min before addition of 200 mM indole-3-acetic acid and 100 mM NH_4_HCO_3_ and incubation at RT for 40 min in the dark. Supernate was removed, gel pieces dried by GeneVac EZ-2, then rehydrated in 13 µg/ml trypsin in 100 mM NH_4_HCO_3_ at 4 °C for 1 h, with additional 100 mM NH_4_HCO_3_ added to submerge gel pieces for overnight incubation at 37 °C. Supernate was transferred to a fresh tube, and excess peptides extracted with addition of 60% acetonitrile/0.1% trifluoroacetic acid and sonication for 15 min in a Sonorex Digitec ultrasonic water bath (Bandelin), repeated three times with solution transferred to a fresh tube each time. Final solution was dried using a GeneVac EZ-2.

### Peptide desalting

Stage-tips were used in this procedure. Tips were washed once with methanol, once with Elution Buffer (80% acetonitrile, 0.1% trifluoroacetic acid) and then three times with Loading Buffer (0.1% trifluoroacetic acid). Peptides were resuspended in Loading Buffer and loaded onto the stage-tip, then washed thrice with Loading Buffer. Peptides were eluted in Elution Buffer, then dried using a GeneVac EZ-2.

### Liquid chromatography tandem mass spectrometry

Peptides were identified using an UltiMate 3000 (Dionex) paired with a Q-Exactive Plus (Thermo Fisher Scientific) in positive polarity mode. Peptides were separated using an in-house packed 75 μm × 40 cm C18AQ column with a 1.9 μm particle size. Separation occurred in a 10–35% ACN gradient containing 0.1% Formic acid at 250 nl/min at 55°C for 90 min. MS1 scan was acquired from a 350–1550 m/z range with a 70 000 resolution, 3e6 automatic gain control (AGC) and 100 ms injection time. Data-dependent acquisition of the top 20 ions was achieved using higher-energy collisional dissociation with a 35 000 resolution, 1e6 AGC, 120 ms injection time, 27% normalised collision energy and a 1.2 m/z isolation width.

### MS data analysis

Raw data was processed using MaxQuant (v1.5.3.24) (2) against a hybrid database, including a complete rat Uniprot database (06/2016, 37 529 entries) (3), one mouse Sorcs1 protein sequence (Uniprot ID Q9JLC4-2) and the corresponding Thr_52_Ile mutant sequence; both containing a C-terminal myc sequence. Processing was conducted using default settings, with the following exceptions: Oxidation of methionine and acetylation of the protein N-terminus were set as variable modifications, and carbaminomethylation of cysteine was set as a fixed modification. Both protein false discovery rate and peptide spectral match were set to 1%. Database-searching was performed twice, with either trypsin or semispecific trypsin as the digestion mode; combining the identification results.

### Statistics

All statistical analysis was performed in GraphPad Prism version 8.2.0. Statistical significance was determined by two-tailed student’s t-test or one-way ANOVA with Tukey’s multiple comparisons post-test. A value of *p* < 0.05 was considered significant.

## Results

### Sorcs1 Thr52lle mutation results in defective basal insulin secretion

**Figure 1:**
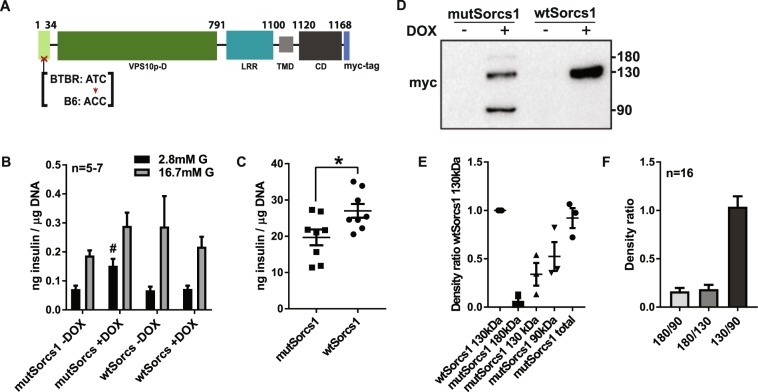
Alternate protein products in *Sorcs1* Thr_52_Ile SNP result in defective insulin secretion. (**A**) Graphical representation of Sorcs1 protein and single-nucleotide polymorphism in pro-domain region. (**B**) Glucose-induced insulin secretion assay performed in *wtSorcs1* and *mutSorcs1*-expressing INS1 cells. Cells were incubated in basal media of 2.8 mM glucose for 1 h, then stimulated at 2.8 mM or 16.7 mM glucose for 1 h. Secreted insulin in media was measured by HTRF assay, then expressed relative to total cellular DNA content. (**C**) Insulin content measured by HTRF normalised to total cellular DNA content of *wtSorcs1* and *mutSorcs1*-expressing INS cells after glucose-stimulated insulin secretion assay. (**D**) Representative western blot of lysates from *wtSorcs1* and *mutSorcs1*-expressing INS1 cells, harvested after 18 h doxycycline (or DMSO control) treatment. Cells were scraped into RIPA buffer containing protease inhibitors, sonicated, then centrifuged to collect lysates. Lysates were run on 7.5% tris-glycine gels for western blotting. (**E**) Densitometry analysis of alternative products observed in myc immunoblot bands in *mutSorcs1*-expressing INS1 cells. (**F**) Densitometry analysis comparing alternate *mutSorcs1* INS bands and *wtSorcs1* bands in myc immunoblot. *p < 0.05 by one-way ANOVA, Tukey’s multiple comparison post-test, ^#^p < 0.05 versus all basal groups by one-way ANOVA, Tukey’s multiple comparison post-test.

It was a Thr_52_Ile mutation in the pro-domain of the mouse *Sorcs1* protein that led to the identification of the gene as a type 2 diabetes gene^10^ (Fig. 1A). We will refer to this allele as *mutSorcs1*. This mutation causes a reduction in insulin levels and congenic mice with insertion of the B6 *mutSorcs1* allele in the BTBR background display lower fasting plasma insulin as well as a reduction in insulin during a glucose tolerance test validating that *mutSorcs1* underlies the associated phenotype^10^. In this study, we assesed the functional consequence of *mutSorcs1 in vitro* by expressing the two variants in INS-1 β-cells and measured glucose-stimulated insulin secretion and insulin content. *MutSorcs1 expressing* INS-1 cells exhibited significantly heightened basal levels of insulin secretion, with an approximate doubling of insulin secretion under basal conditions (p < 0.05 by one-way ANOVA), and reduced glucose-stimulated insulin secretion (Fig. 1B). Further, cellular insulin content of *mutSorcs1* expressing INS-1 cells was significantly lower compared to cells expressing *wtSorcs1* (Fig. 1C), validating that our cell system phenocopies the loss of insulin associated with the mutation in mice.

### Sorcs1 Thr52lle mutation forms an uncleaved protein product of 90 kDa

*Sorcs1* is translated as a precursor protein (pro-Sorcs1) which is processes to its mature 130 kDa form by furin-mediated cleavage of an N-terminal 77 animo acids pro-peptide^15^. We obtained a clue to the functional consequence of *mut*Sorcs1 when we expressed the two variants that contained C-terminal myc-tag in INS1 β-cells, under a doxycycline-inducible system. On 7.5% SDS-PAGE, immunoblotting of the myc-tag showed that *wtSorcs1* allele expression results in the formation of a large band at 130 kDa corresponding to the mature protein. However, expression of the Thr_52_Ile *mutSorcs1* allele was visualised as three unique bands corresponding to not only the 130 kDa mature protein band, but additional bands at approximately 90 kDa and 180 kDa (Fig. 1D). It was likely that the large band observed at 130 kDa also contained the pro-form of the Sorcs1 (pro-Sorcs1), and the mature 130 kDa band of both *wtSorcs1* and *mutSorcs1* resolved as a doublet containing pro-Sorcs1 and mature Sorcs1 in an 7.5% SDS-PAGE when run for an extended duration to specifically separate out higher density proteins (Supplementary Fig. 1A). This was also observed when Sorcs1 was separated on a Bolt™ 4–12% Bis-Tris Plus Gels in Bolt MOPS SDS running buffer (Supplementary Fig. 1B). Densitometry analysis of the *mutSorcs1* bands demonstrated that their cumulative density was similar to that of the 130 kDa *wtSorcs1* band (Fig. 1E), while densitometry analysis of multiple *mutSorcs1* samples indicated that the 90 kDa and 130 kDa bands constituted the predominant forms of the protein (Fig. 1F). We hypothesised that a processing defect, such as additional N-terminal cleavage of *mutSorcs1* (as both forms possess the c-terminal myc-tag), could be responsible for the lighter protein product.

### Additional 90 kDa mutSorcs1 band is not altered by proprotein cleavage

**Figure 2:**
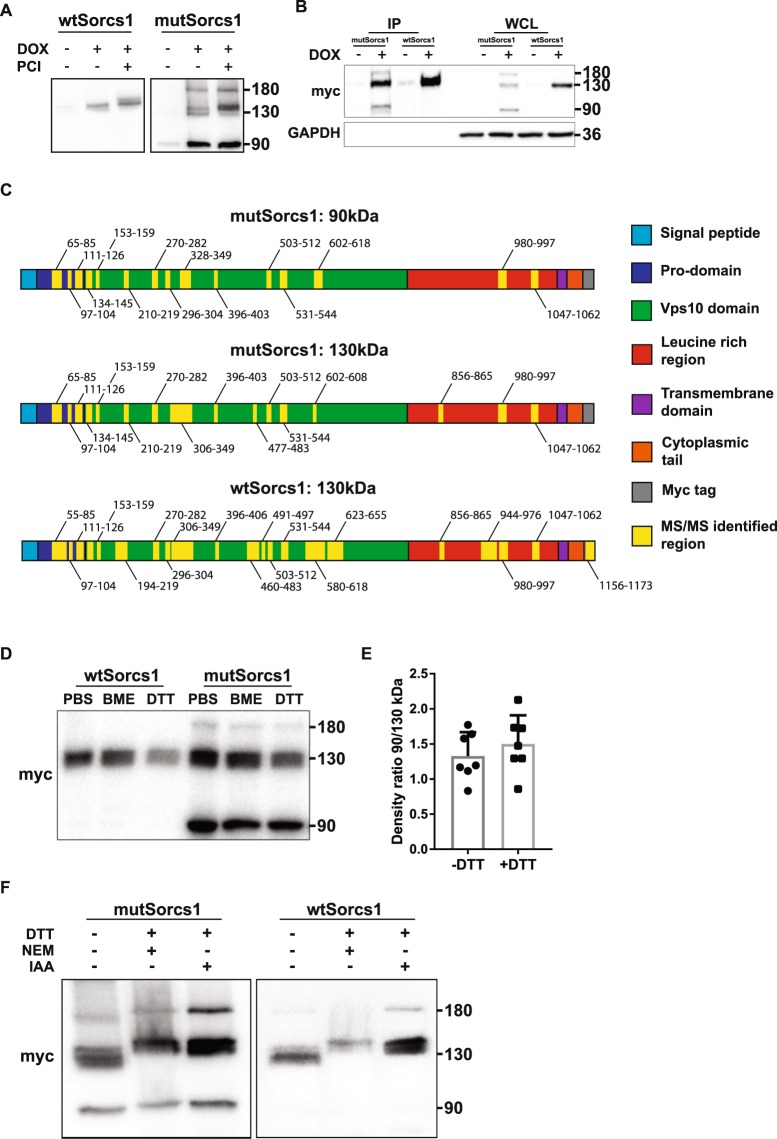
The alternative 90 kDa protein product of mutSorcs1 is unaffected by pro-protein convertases. (**A**) Representative western blot of lysates from *wtSorcs1* and *mutSorcs1*-expressing INS1 cells treated with DMSO or 10 µM prohormone convertase inhibitor (PCI). (**B**) Immunoprecipitation of Sorcs1 from *wtSorcs1* and *mutSorcs1*-expressing INS1 whole cell lysates (WCL) using anti-myc tag antibody-conjugated agarose beads. (**C**) Representative peptide coverage of 130 kDa and 90 kDa bands excised from SDS-PAGE of *wtSorcs1* and *mutSorcs1-*expressing INS1 cells and subject to LC-MS/MS data analysis. (**D**) Representative western blot of *wtSorcs1* and *mutSorcs1*-expressing INS1 cells in reducing and non-reducing conditions. (**E**) Densitometry analysis of *mutSorcs1* bands immunoblotted in the presence or absence of 100 mM dithiothreitol (DTT). (**F**) Representative western blot of *wtSorcs1* and *mutSorcs1*-expressing INS1 cells in reducing conditions in the presence of 50 mM N-Ethylmaleimide (NEM) or iodoacetamide (IAA).

Conversion of pro-Sorcs1 to its mature form normally occurs through processing by furin-mediated cleavage at two sites^15^, however, an additional pro-protein convertase cleavage site was predicted to exist in both *Sorcs1* alleles^16^. As the 90 kDa *mutSorcs1* band retained its C-terminal myc tag, we aimed to investigate if the *mutSorcs1* protein was the result of additional cleavage sites. To investigate this, both *wtSorcs1* and *mutSorcs1* - expressing INS-1 cells were treated with a cell permeable pro-protein convertase inhibitor (PCI) targeted to furin, but also PC1/3, PC4, PC5/6 and PACE4^17^ in a dose curve (Supplementary Fig. 2A). SDS-PAGE of control and PCI treated cells revealed a small shift in the 130 kDa band in both *wtSorcs1* and *mutSorcs1*–expressing cells to a slightly heavier product at a 10 µM treatment dose, corresponding to a density shift to the pro-Sorcs1 protein (Fig. 2A). A specific furin-inhibitor also mediated the same shift in the 130 kDa wtSorcs1 band (Supplementary Fig. 2B). However, no change in mass was observed in either the lighter 90 kDa fragment or the heavier 180 kDa fragment of Sorcs1 resulting from expression of the *mutSorcs1* allele, indicating that its generation was unaffected by pro-protein convertase activity.

To more definitively characterise the *mutSorcs1* 90 kDa product, peptide identification by LC-MS/MS was performed on 130 kDa and 90 kDa protein bands excised from gels processed from immunoprecipitated Sorcs1 in *wtSorcs1* and *mutSorcs1* – expressing INS1 samples (Fig. 2B). As expected, the 130 kDa band (corresponding to both pro-Sorcs1 and mature Sorcs1) products from *wtSorcs1* and *mutSorcs1* demonstrated similar peptide coverage. Most strikingly, peptide coverage of the *mutSorcs1* 90 kDa product also spanned coverage of the pro-domain of the Sorcs1 protein (Fig. 2C). In agreement with the PCI experiments, these data demonstrate that the protein size discrepancy is not a result of N-terminal cleavage of the mutant protein. Raw peptide coverage data for all three experiments are included in Supplementary Table 1, and peptide quantification by trypsin and semi-specific trypsin digestion excluded peptide contamination.

### Additional 90 kDa mutSorcs1 band is not altered by disulphide bonds

All available cysteines within the common Vps10p-D of Sortilin, including 10 of the conserved cysteines within the C-terminal region, have been shown to form disulphide bonds^18^. Using prediction software analysis, Sorcs1 is expected to have 10 high confidence disulphide bonds. Therefore, we tested the possibility that altered intermolecular disulphide bonding was the cause of the additional *mutSorcs1* protein bands observed. INS1 cells expressing *wtSorcs1* and *mutSorcs1* were immunoblotted in both reducing and non-reducing conditions, however the migration of all *mutSorcs1* protein bands were unchanged (Fig. 2D). Densitometry analysis also found no difference between density ratios of the 130 kDa and 90 kDa *mutSorcs1* bands in reducing and non-reducing conditions (Fig. 2E).

As the reducing agents used above (DTT and 2-mercaptoethanol) were potentially reversible, we validated these findings by performing irreversible blocking of cysteine oxidation with iodoacetamide or NEM under reducing conditions. A small shift in motility of all *mutSorcs1* bands, as well as the 130 kDa *wtSorcs1* band (Fig. 2F) indicated that all protein products contained oxidized cysteines – though their presence in the 90 kDa *mutSorcs1* was not enough to account for the molecular mass discrepancy compared to *wtSorcs1*. To eliminate potential misinterpretation of data due to reagents diffusing between adjacent lanes during SDS-PAGE, we included blank lanes between reduced versus non-reduced samples and obtained similar results (Supplementary Fig. 2C).

### The 90 kDa mutSorcs1 protein product is formed and retained in the ER

**Figure 3:**
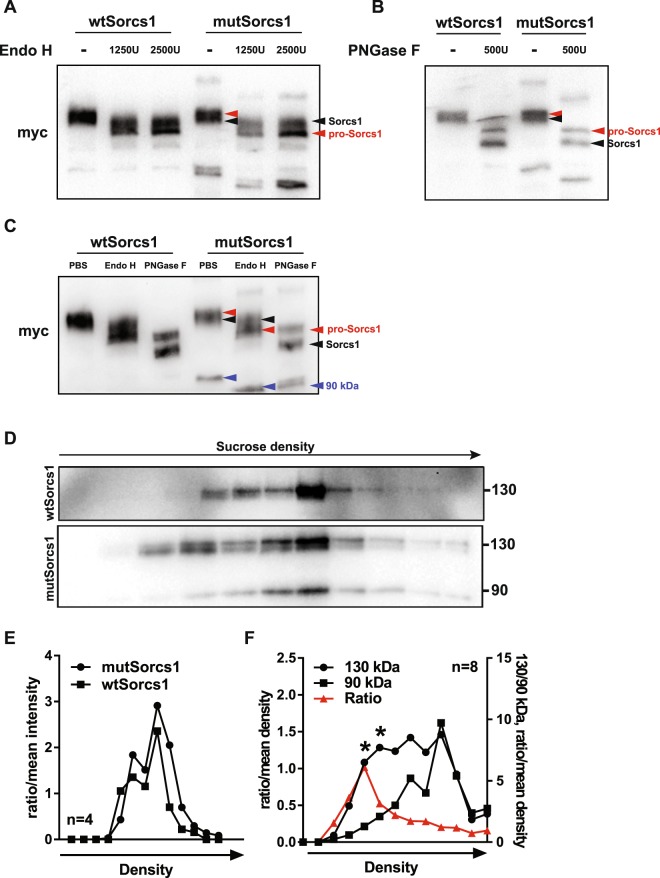
The alternative 90 kDa protein product exhibits a different glycosylation profile. (**A**) Representative western blot of *wtSorcs1* and *mutSorcs1* INS1 cells after de-glycosylation treatment with 1250 U or 2500 U Endo H. (**B**) Representative western blot of *wtSorcs1* and *mutSorcs1* INS1 cells after de-glycosylation treatment with 500 U PNGase F. (**C**). Western blot of *wtSorcs1* and *mutSorcs1* INS1 cells after de-glycosylation treatment with 1250 U Endo H and 500 U PNGase F. (**D**) Representative western blot of subcellular fractionation by sucrose gradient of *wtSorcs1* and *mutSorcs1*-expressing INS1 cells. (**E**) Densitometry analysis of combined bands in subcellular fractionation immunoblots in *wtSorcs1* and *mutSorcs1* INS1 cells. (**F**) Densitometry analysis of 180, 130 and 90 kDa *mutSorcs1* INS1 cell bands in subcellular fractionation immunoblots. *p < 0.05, unpaired t-test of ratio/mean density of 130 kDa compared to 90 kDa band in Fraction 5 and Fraction 6

Sequence analysis of murine *Sorcs1* predicted the presence of eight *N*-linked glycans^19^. As such, alternative post-translational glycosylation in *mutSorcs1* was investigated as the cause of the 90 kDa product. INS1 cells expressing *wtSorcs1* and *mutSorcs1* were subjected to glycan cleavage by either Endoglycosidase H (Endo H) – which specifically cleaves high-mannose *N*-linked glycans, or Peptide-*N*-Glycosidase F (PNGase F) – which cleaves almost all *N-*linked glycans. Each deglycosylation reaction demonstrated distinct mobility shifts for pro-Sorcs1 and mature Sorcs1 bands in both *wtSorcs1 and mutSorcs1* lysates. As expected, pro-Sorcs1 (red arrows) – but not processed mature Sorcs1 (black arrows) – shifted to a lower molecular weight when exposed to Endo H, consistent with its localisation within the ER as a uncleaved pro-peptide (Fig. 3A). PNGase F decreased the mass of pro-Sorcs1, and also mature Sorcs1, corresponding with the increasingly de-glycosylated Sorcs1 protein (Fig. 3B). Of significant interest is that the band shifts apparent in both the pro-Sorcs1 protein and 90 kDa *mutSorcs1* product were identical in both Endo H treatment and PNGase F treatment (red and blue arrows, Fig. 3C), indicating the presence of only high-mannose *N-*linked glycans in these proteins – and a lack of Endo H resistance. This data suggests that, like pro-Sorcs1, the 90 kDa mutSorcs1 protein is ER localised.

To investigate this further we performed subcellular fractionation analysis of INS-1 cells expressing either the *wtSorcs1* or *mutSorcs1* allele to identify potential changes in subcellular localisation (Fig. 3D). Total densities of *wtSorcs1* and *mutSorcs1* bands co-fractionated almost identically (Fig. 3E), however, comparison of the two *mutSorcs1* bands identified subtle differences in subcellular localisation between the 130 kDa *wtSorcs1-*corresponding band and the 90 kDa band, with peak densities of the latter appearing in heavier sucrose fractions (Fig. 3F).

**Figure 4:**
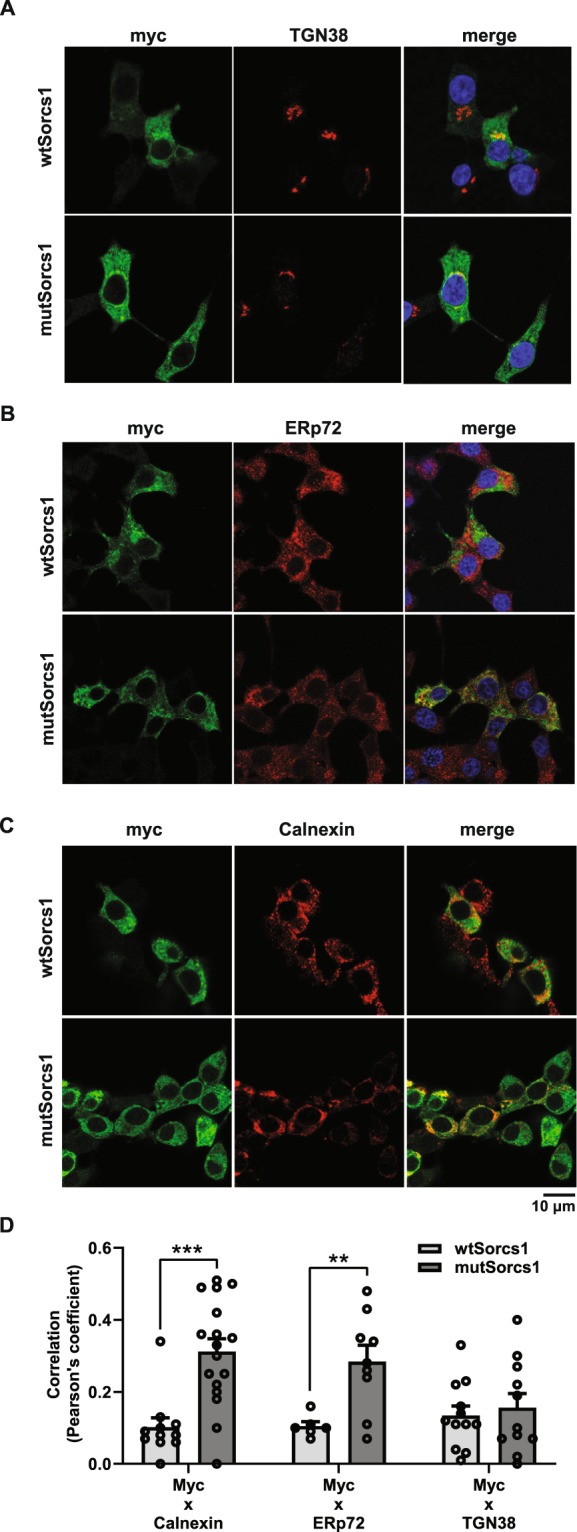
The alternative 90 kDa protein product has different localisation to the mature protein. (**A**) Representative confocal images from immunofluorescent staining of myc and TGN38 in *wtSorcs1* and *mutSorcs1*-expressing INS1 cells. (**B**) Representative confocal images from immunofluorescent staining of myc and ERp72 in *wtSorcs1* and *mutSorcs1*-expressing INS1 cells. (**C**) Representative confocal images from immunofluorescent staining of myc and Calnexin in *wtSorcs1* and *mutSorcs1*-expressing INS1 cells. Representative images were selected from 6–10 Z-stack images, containing 1–4 cells per image, across two separate experiments. (**D**) Peasons coefficient colocalisation analysis of myc and Calnexin, myc and ERp72 and myc and TGN38 in *wtSorcs1* and *mutSorcs1*- expressing cells averaged from 6–17 images across two separate experiments. ***p < 0.001, **p < 0.01 by unpaired t-test.

To further investigate Sorcs1 subcellular distribution, immunofluorescent staining of myc and insulin was performed in both myc-tagged *wtSorcs1* and *mutSorcs1* expressing INS1 cells. Myc was initially co-stained with Sorcs1 to confirm significant co-localisation, with both appearing in reticular structures in the cell periphery (Supplementary Fig. 3A). Insulin co-staining with myc demonstrated no obvious co-localisation between Sorcs1 and insulin granules, in either *wtSorcs1* or *mutSorcs1* (Supplementary Fig. 3B), though insulin granules appeared characteristically punctate in the cell periphery, with increased intensity in perinuclear regions likely corresponding to pro-insulin (or immature insulin granules) in the TGN. As we previously reported^14^, myc/Sorcs1 demonstrated co-localisation with the TGN marker TGN38, notably here in both *wtSorcs1* and *mutSorcs1* INS1 cells (Fig. 4A), however only *mutSorcs1* INS1 cells displayed consistent partial co-localisation with the ER markers ERp72 or Calnexin (Fig. 4B,C). Quantification by Pearsons correlation coefficient demonstrated significantly increased co-localisation of ER markers in *mutSorcs1*-expressing cells than *wtSorcs1*-expressing cells, but no difference in TGN38 co-localisation (Fig. 4D, p < 0.01).

### MutSorcs1 90 kDa exhibits a different half life to the wildtype protein

**Figure 5:**
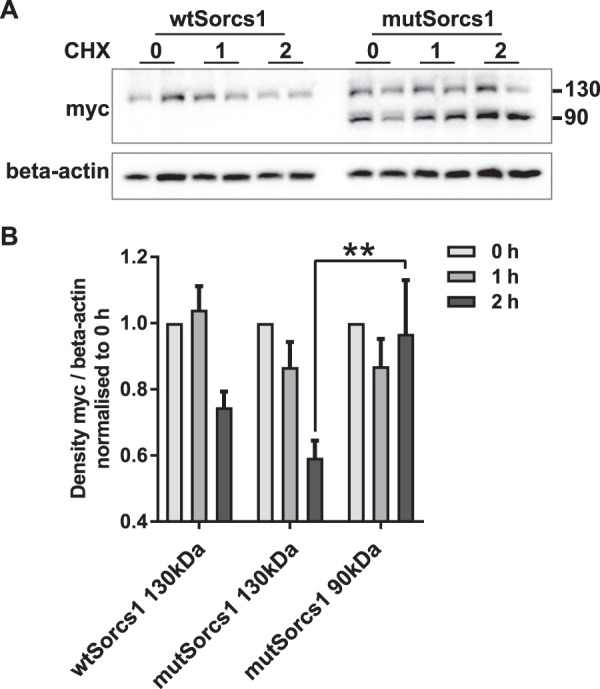
The alternative 90 kDa protein product has an increased half-life. (**A**) Representative western blot of *wtSorcs1* and mutSorcs1-expressing cells after DMSO or 5 µM cyclohexamide (CHX) treatment for 1 and 2 hours. (**D**) Densitometry analysis of 130 kDa and 90 kDa bands in cyclohexamide treatment of *wtSorcs1* and *mutSorcs1*-expressing cells. **p < 0.01 by one-way ANOVA, Tukey’s multiple comparison post-test.

As these data lend evidence to the presence of a differentially modified, ER-localised mutant Sorcs1 product, we treated *wtSorcs1* and *mutSorcs1*-expressing INS1 cells with cyclohexamide to investigate protein degradation. We found that *mutSorcs1*-expressing cells demonstrated increased retention of the mutant 90 kDa protein 2 hours post-treatment compared to the 130 kDa protein product (p < 0.01, Fig. 5A,B), further validating an alternate processing pathway for the mutant form protein associated with the Thr_52_Ile single nucleotide polymophism in *Sorcs1*.

## Discussion

Natural genetic variations can offer better mechanistic insights into complex biological processes that are more informative than investigations using complete gene deletion. This is because a mutation in a gene can either be a loss or gain-of-function mutation. The objective of this study was to elucidate the functional consequence of the Thr_52_Ile single nucleotide polymorphism in the pro-domain of the *Sorcs1* gene that has been associated with type 2 diabetes. Expression of the mutant allele of *Sorcs1* in INS1 cells clearly demonstrated alternative processing of the Sorcs1 protein compared to wildtype *Sorcs1* when visualised by SDS-PAGE. The significantly lower molecular mass of the mutant protein was striking. The formation of an additional protein band of 90 kDa initially suggested that an additional cleavage of *mutSorcs1* by a protease such as furin, however pro-protein convertase inhibition resulted in no change in the size of the alternative band. Furthermore, an upward band shift of the mature 130 kDa band indicated that there was normally efficient processing of the mature Sorcs1 protein from the pro-form in both *mutSorcs1* and *wtSorcs1 -* expressing INS1 cells.

It appears that the 90 kDa product of *mutSorcs1* protein is misfolded, though we have been unable to definitively confirm this in our current study. Despite at least 10 cysteine sites at which intramolecular disulphide bonds were predicted to occur, irreversible blocking of cysteine oxidation eliminated a role for disulphide bonds as a source for alternatively folded *mutSorcs1* protein products. The retention of the pro-domain as identified by LC-MS/MS, and high-mannose glycosylation profile indicates that this 90 kDa product of *mutSorcs1* protein does not completely access the TGN – in which furin-mediated pro-domain cleavage, and hybrid or complex glycosylation occurs. Subcellular fractionation of INS1 further supports the notion that the additional *mutSorcs1* protein band localises differently to the 130 kDa *wtSorcs1* protein band and therefore undergoes differential processing through subcellular compartments. Immunofluorescent staining also demonstrated ERp72, calnexin and myc co-staining primarily in *mutSorcs1* INS1 cells and not in *wtSorcs1* INS1 cells, with TGN38 co-localisation in both cell lines. Finally, the 90 kDa mutSorcs1 protein exhibits an increased half life compared to the 130 kDa protein, providing further evidence for its mis-localisation and abnormal trafficking within the cell.

From a functional perspective, earlier work with congenic mouse strains, we found that *mutSorcs1* allele in the BTBR mouse background showed impaired insulin secretion in response to an intraperitonial glucose challenge as well as a 30% reduction in fasting plasma insulin relative to the BTBR mouse^10^. We hypothesize that one explanation for this observation is that insertion of *mut*Sorcs1 into the BTBR mouse strain background favors the formation of the misfolded Sorcs1 protein to a greater extent than we observed in the INS1 β-cell line.

Our data suggests that the alternatively processed or misfolded *mutSorcs1* products accumulate in the ER and reduce the expression of mature 130 kDa Sorcs1 protein which is a major determinant of insulin content. This reduction in mature 130 kDa *Sorcs1*, reduces normal β-cell function. The increased insulin secretion at 2.8 mM glucose observed in *mutSorcs1* INS1 cells is also reminiscent of the elevated basal (pro)insulin secretion characteristic of Type 2 Diabetes, and indicative of dysregulated proximal insulin processing. Though these data are incongruent with the reduced plasma insulin levels seen in B6 *Sorcs1* allele-expressing mice^10^, it is likely that the elimination of whole-body Sorcs1 is incomparable with the more subtle phenotype of a single nucleotide polymorphism. Pro-peptide cleavage in the TGN of VPS10 proteins is required for exposure of ligand-binding regions in both Sortilin^20^ and SorLA, and these pro-peptides are then capable of binding to its own mature protein^18^. This is hypothesised to be a form of self-regulation by either assisting with protein-folding or to inhibit premature ligand binding^18^. While Sorcs1 pro-peptide has low affinity for mature Sorcs1, it instead is capable of binding mature Sortilin^15^. Sorcs1 has thereby been implicated in post-Golgi cargo trafficking, primarily through its inhibition of Sortilin-regulated ligand uptake and transcription factor interaction by competitive binding of mature Sortilin protein^15^. As such, it stands to reason that a 50% reduction of the mature 130 kDa Sorcs1 protein may in turn result in an increase of Sortilin function. This is further evidenced in INS1 cells overexpressing Sortilin, which exhibit decreased total insulin content (unpublished observations), implicating a role for Sortilin in the downregulation of insulin within the β-cell, potentially through its role in lysosomal trafficking^21,22^. However, although Sortilin is a known receptor for soluble ligands such as amyloid precursor protein^23^, neurotensin^24^, PCSK9^25^, as well as being involved in retrograde trafficking of GLUT4^26^, it is not yet known whether sortilin is also capable of regulating proinsulin trafficking.

Polymorphisms affecting Sorcs1 function have previously been reported^7,12,27^. Similarly, pro-domain missense mutations are not uncommonly associated with disease^28,29^. We have demonstrated that the Thr_52_Ile mutant Sorcs1 product is subject to impaired processing in the β-cell. Sorcs1 trafficking and function in particular, appears highly regulated by its pro-domain. We speculate that aberrant function of β-cells associated with Thr_52_Ile mutation in the *Sorcs1* gene is driven by the stoichiometric loss of wildtype Sorcs1 protein in favour of the mutant form of the protein. This, in turn, may imbalance the trafficking network associated with normal insulin content and secretory function.

## Supplementary information

Supplementary Data

## Data Availability

The datasets generated during and/or analysed during the current study are available from the corresponding author on reasonable request.
