# Supplementary material for: Type 2 diabetes-associated single nucleotide polymorphism in Sorcs1 gene results in alternative processing of the Sorcs1 protein in INS1 β-cells
Source: Sci Rep. 2019 Dec 19;9:19466. doi: 10.1038/s41598-019-55873-6 (PMC6923373; doi:10.1038/s41598-019-55873-6)
Supplement: Supplementary file 1 — Supplementary Data [file 41598_2019_55873_MOESM1_ESM.pdf]

Type 2 diabetes-associated single nucleotide polymorphism in Sorcs1 gene results in alternative processing of the Sorcs1 protein in INS1 β-cells.

Belinda Yau, Zachary Blood, Yousun An, Zhiduan Su and Melkam A Kebede*.

Charles Perkins Centre, School of Life and Environmental Sciences, Faculty of Science, The University of Sydney, Sydney, New South Wales, Australia.

Supplementary Figure 1. (A) Representative western blot of two replicate *wtSorcs1* and *mutSorcs1*-expressing cell samples (1 and 2) on a 7.5% SDS-PAGE with extended run-time, showing doublet band at 130 kDa of pro-Sorcs1 and mature Sorcs1. (B) Western blot of *wtSorcs1* and *mutSorcs1*-expressing cell samples on a Bolt™ 4-12% Bis-Tris Plus Gels in Bolt MOPS SDS running buffer, showing doublet band at 130 kDa of pro-Sorcs1 and mature Sorcs1.

Supplementary Figure 2. (A) Representative western blot of lysates from *wtSorcs1* and *mutSorcs1*-expressing INS cells treated with DMSO or 15 pM, 10 mM, 100mM or 10 µM prohormone convertase inhibitor (PCI). (B). Representative western blot of lysates from *wtSorcs1* and *mutSorcs1*-expressing INS1 cells treated with DMSO, 10 µM prohormone convertase inhibitor, or 30 µM furin inhibitor. (C). Representative western blot of *wtSorcs1* and *mutSorcs1*-expressing INS cells in non-reducing and reducing conditions in the presence of N-Ethylmaleimide (NEM) or iodoacetamide (IAA).

Supplementary Figure 3. (A) Representative images from immunofluorescent staining of myc and Sorcs1 in *wtSorcs1* and *mutSorcs1*-expressing INS cells. (B) Representative images from immunofluorescent staining of myc and insulin in *wtSorcs1* and *mutSorcs1*-expressing INS cells.

Supplementary Table 1. List of identified peptides (as amino acid ranges) in 3 unique LC-MS/MS runs from anti-myc immunoprecipitation samples of 130 kDa *wtSorcs1* and 130 kDa and 90 kDa *mutSorcs1* gel-excised bands.

Supplementary Figure 1

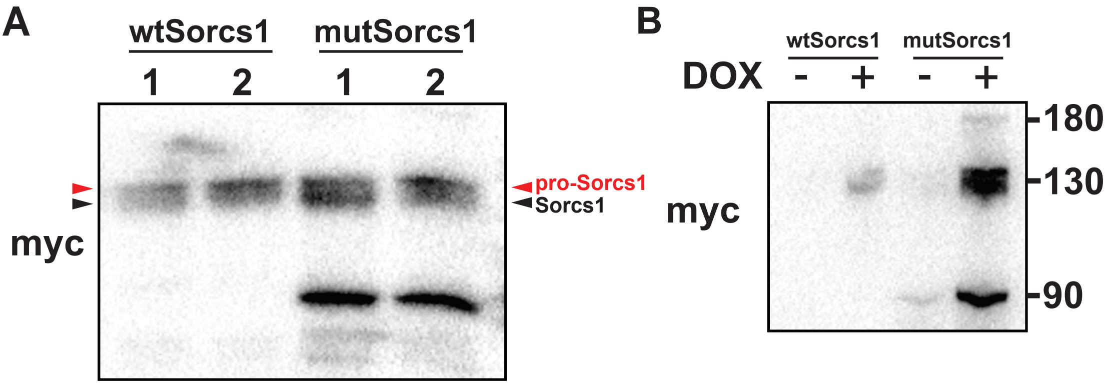

Supplementary Figure 2

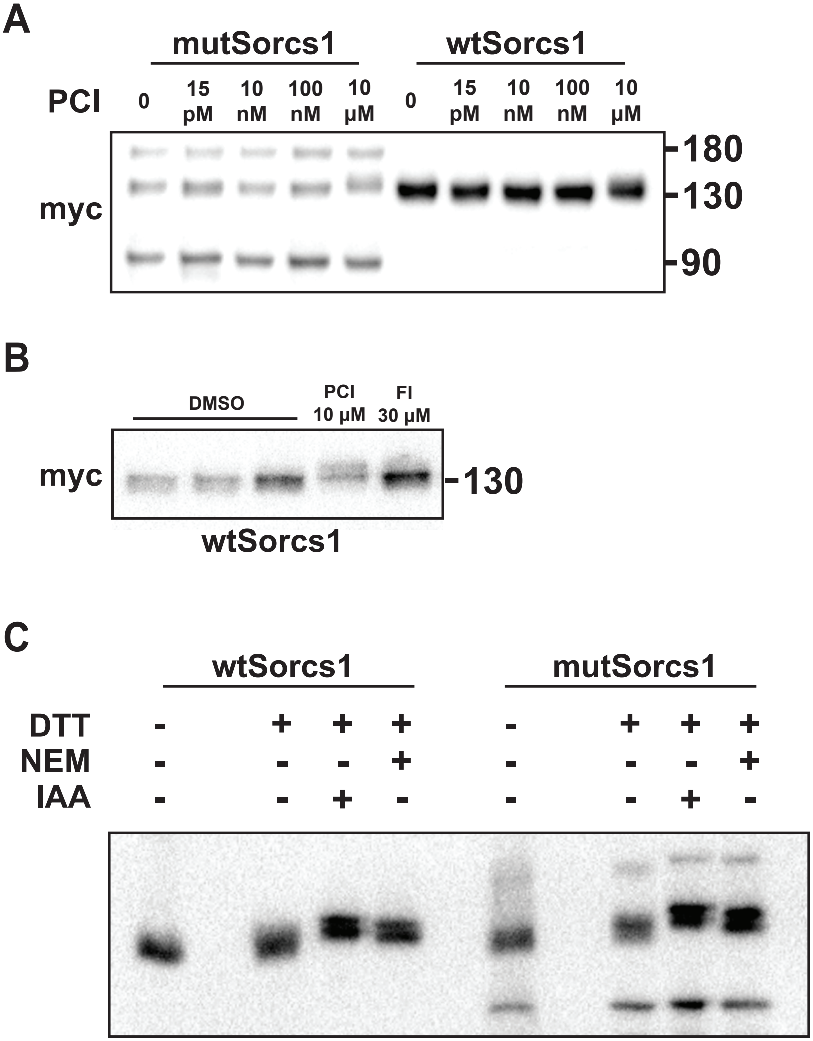

Supplementary Figure 3

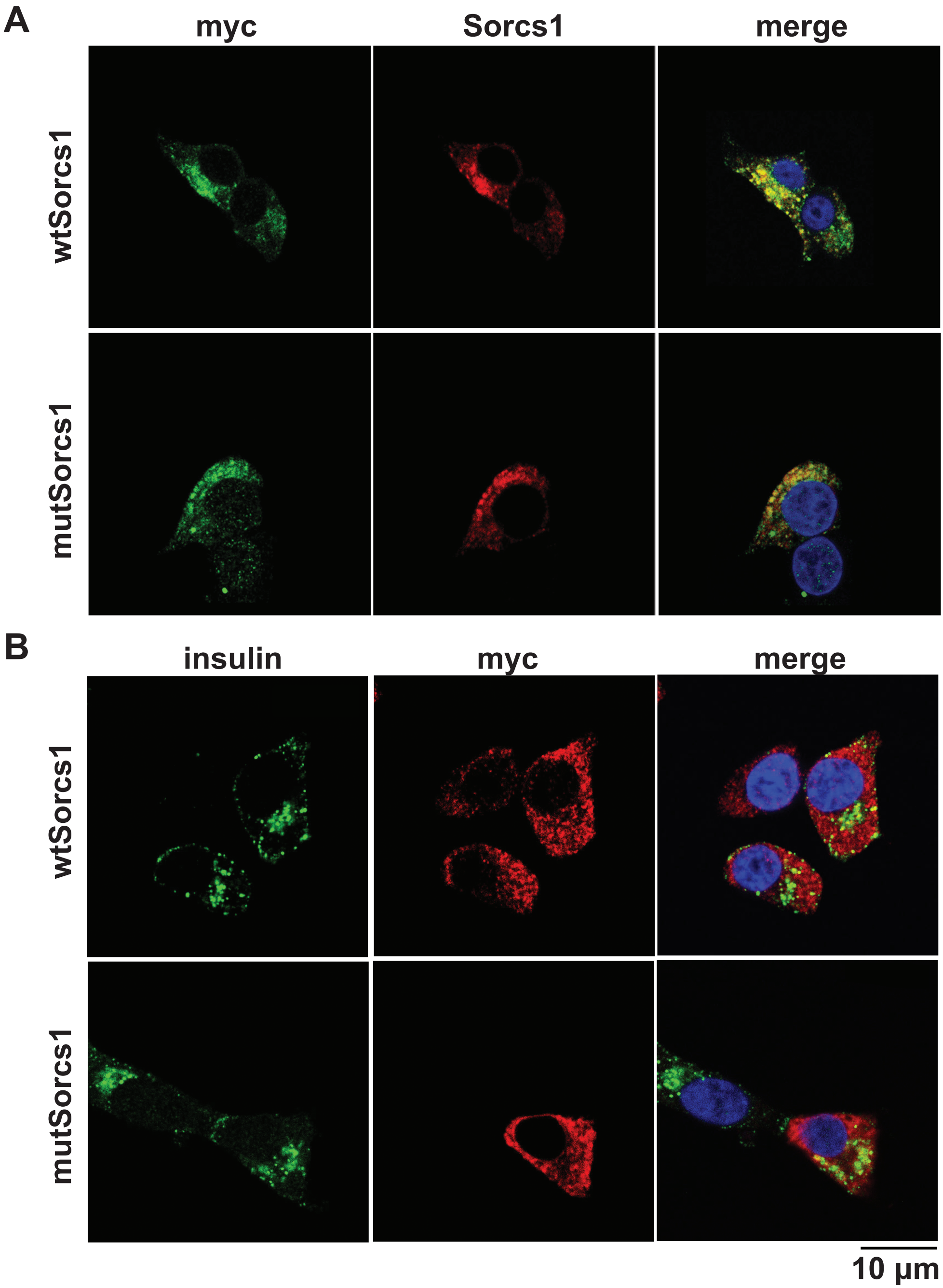

Supplementary Table 1

	Amino acid ranges identified; pro-peptide aa highlighted		
	mutSorcs1 90 kDa	mutSorcs1 130 kDa	wtSorcs1 130 kDa
Run1	134 – 145	55 – 85	76 – 87
	210 – 220	97 – 104	97 – 104
	503 – 512	111 – 126	133 – 145
	602 - 608	133 – 145	194 – 219
		194 – 219	317 – 349
		270 – 282	503 – 512
		295 – 349	531 – 544
		387 – 403	602 – 617
		477 – 483	981 – 997
		491 – 497	1047 – 1062
		531 – 544	
		580 – 601	
		608 – 617	
		855 – 864	
		981 – 997	
		1046 – 1070	
Run 2	65 – 85	97 – 104	65 - 85
	97 – 104	111 – 126	97 – 104
	111 – 126	295 – 304	111 – 126
	133 – 145	503 – 512	133 – 145
	153 – 159	531 – 544	153 – 159
	210 – 219	602 – 608	210 – 219
	270 – 282		270 – 282
	295 – 304		305 – 349
	328 – 349		396 – 403
	396 – 403		477 – 483
	503 – 512		503 – 512
	531 – 544		531 – 544
	602 – 617		602 – 608
	981 – 997		855 – 864
	1046 – 1070		981 – 997
			1046 – 1070
Run 3	86 – 92	34 – 48	111 – 126
	97 – 104	97 – 104	133 – 145
	111 – 126	111 – 126	295 – 304
	210 – 219	130 – 151	328 – 337
	295 – 304	210 – 219	503 – 512
	328 – 337	228 – 240	531 – 544
	491 – 497	295 – 304	580 – 608
	503 – 512	396 – 413	855 – 864
	531 – 544	477 – 483	944 – 976
	602 – 608	491 – 497	981 – 997
		503 – 512	1046 – 1070
		531 – 544	
		602 – 608	
		855 – 864	
		968 – 976	
